# Supplementary material for: Subthreshold moment analysis of neuronal populations driven by synchronous synaptic inputs
Source: ArXiv. 2025 Mar 17:arXiv:2503.13702v1. Preprint. [Version 1] (PMC11957229)
Supplement: Supplement 1 [file NIHPP2503.13702v1-supplement-1.pdf]

Supporting information

S1 Appendix. Formulas for the shifted moments. Let us consider a set of feedforward population of neurons A. For all $a \in A$, we denote the shifted membrane voltage of neuron a by $U_a = V_a - I_a/G_a$. Given a multiset $B = \{a_1, \dots, a_n\}$ of neuron indices in A, our goal here is to compute the shifted stationary moments

$$\mu_B = \mathbb{E}\left[\prod_{a \in B} U_a\right] .$$

To this end, let us adopt the shorthand notations,

$$Q_a = R_a - I_a/G_a \,, \quad R_a = \frac{W_{\mathrm{e},a} V_\mathrm{e} + W_{\mathrm{i},a} V_\mathrm{i}}{W_{\mathrm{e},a} + W_{\mathrm{i},a}} \,, \quad Y_a = e^{-(W_{\mathrm{e},a} + W_{\mathrm{i},a})} \,.$$

Combining the population-level Marcus update rule Eq. (10) and the time shift-invariance of the Palm

distribution Eq. (11), the PASTA principle yields

$$\begin{aligned}
\mu_B &= \mathbb{E}\left[\prod_{a\in B}\left((U_a+J_a)e^{-\frac{S_1}{\tau_a}}\right)\right] , \\
&= \mathbb{E}\left[e^{-\sum_{a\in B}\frac{S_1}{\tau_a}}\prod_{a\in B}\left(U_a+(Q_a-U_a)\left(1-Y_a\right)\right)\right] , \\
&= \mathbb{E}\left[e^{-\sum_{a\in B}\frac{S_1}{\tau_a}}\right]\mathbb{E}\left[\prod_{a\in B}\left(U_aY_a+Q_a\left(1-Y_a\right)\right)\right] ,
\end{aligned}$$

where the last equation follows from the fact that inter-arrival times are independent from the driven processes. Utilizing the fact that S_1 is exponentially distributed with rate b, we further obtain by integration that

$$\begin{aligned}
\mu_B &= \left(1+\sum_{a\in B}\frac{1}{b\tau_a}\right)^{-1}\mathbb{E}\left[\prod_{a\in B}\left(U_aY_a+Q_a\left(1-Y_a\right)\right)\right] , \\
&= \left(1+\sum_{a\in B}\frac{1}{b\tau_a}\right)^{-1}\sum_{C\subset B}\mathbb{E}\left[\left(\prod_{a\in C}U_aY_a\right)\left(\prod_{a\in B\setminus C}Q_a\left(1-Y_a\right)\right)\right] , \\
&= \left(1+\sum_{a\in B}\frac{1}{b\tau_a}\right)^{-1}\sum_{C\subset B}\mathbb{E}\left[\prod_{a\in B}U_a\right]\mathbb{E}\left[\prod_{a\in C}Y_a\prod_{a\in B\setminus C}Q_a\left(1-Y_a\right)\right] , \qquad (27)
\end{aligned}$$

where the last equation follows from the fact that the jumps are independent from the driven processes. In order to evaluate the shifted stationary moment μ_B, it is then convenient to introduce the following expectations as coefficients

$$\gamma_{B,C} = \mathbb{E}\left[\prod_{a\in C}Y_a\left(\prod_{a\in B\setminus C}Q_a\left(1-Y_a\right)\right)\right] ,$$

for all multisets $C\subset B$ of indices in A. Then, recognizing the shifted moments in the right-hand side of Eq. (27), the moments μ_B can be determined as the solutions of a triangular system of equations

$$\mu_B = \left(1+\sum_{a\in B}\frac{1}{b\tau_a}\right)^{-1}\sum_{C\subset B}\gamma_{B;C}\mu_C . \qquad (28)$$

Upon solving for μ_B, the above triangular system leads to the following recursive specification for the moments

$$\mu_B = \frac{1}{\alpha_B}\left(\sum_{C\subsetneq B}\gamma_{B;C}\mu_C\right) ,$$

where the coefficient α_B is given by

$$\alpha_B = 1-\mathbb{E}\left[\prod_{a\in B}Y_a\right]+\sum_{a\in B}\frac{1}{b\tau_a} . \qquad (29)$$

Recursively applying the above formula yields an explicit form for the shifted moments

$$\mu_B = \sum_{m=1}^{n}\sum_{\varnothing=B_0\subsetneq\ldots\subsetneq B_m=B}\prod_{k=1}^{m}\left(\frac{\gamma_{B_k,B_{k-1}}}{\alpha_{B_k}}\right) . \qquad (30)$$

S2 Appendix. First-order moments. Applying Eq. (45) for $A_1 = \{a\}$ yields the first-order shifted moments as

$$\mu_a = \mu_{\{a\}} = \frac{\gamma_{\{a\},\varnothing}}{\alpha_{\{a\}}} = \frac{\mathbb{E}\left[Q_a(1-Y_a)\right]}{1/(b\tau_a) + \mathbb{E}\left[1-Y_a\right]} = \frac{\mathbb{E}\left[\left(R_a - I_a/G_a\right)(1-Y_a)\right]}{1/(b\tau_a) + \mathbb{E}\left[1-Y_a\right]} .$$

An alternative and useful expression for μ_a is obtained by observing that

$$\mathbb{E}\left[Q_a(1-Y_a)\right] = \mathbb{E}\left[(R_a - m_a)(1-Y_a)\right] + \mu_a \mathbb{E}\left[1-Y_a\right] .$$

so that we have

$$\mu_a = b_a \tau_a \mathbb{E}\left[(R_a - m_a)(1-Y_a)\right] . \tag{31}$$

Remembering that $\mu_a = m_a - I_a/G_a$, we deduce that the mean voltage m_a satisfies

$$m_a = \frac{b\tau_a \mathbb{E}\left[R_a\left(1-Y_a\right)\right] + I_a/G_a}{1 + b\tau_a \mathbb{E}\left[1-Y_a\right]} = \frac{c_{\mathrm{e},a} V_{\mathrm{e},a} + c_{\mathrm{i},a} V_{\mathrm{i},a} + I_a/G_a}{1 + c_{\mathrm{e},a} + c_{\mathrm{i},a}} ,$$

where the coefficients $c_{\mathrm{e},a}$ and $c_{\mathrm{i},a}$ are given by

$$\begin{aligned} c_{\mathrm{e},a} &= b\tau_a \mathbb{E}\left[\frac{W_{\mathrm{e},a}}{W_{\mathrm{e},a} + W_{\mathrm{i},a}}\left(1 - e^{-(W_{\mathrm{e},a} + W_{\mathrm{i},a})}\right)\right] , \\ c_{\mathrm{i},a} &= b\tau_a \mathbb{E}\left[\frac{W_{\mathrm{i},a}}{W_{\mathrm{e},a} + W_{\mathrm{i},a}}\left(1 - e^{-(W_{\mathrm{e},a} + W_{\mathrm{i},a})}\right)\right] . \end{aligned} \tag{32}$$

S3 Appendix. Generic formulas for the mixed central moments. Our goal here is to derive an characterization of the centered moments

$$M_B = \mathbb{E}\left[\prod_{a \in B} (V_a - m_a)\right] , \tag{33}$$

from the recursive definition of the shifted moment μ_B given by Eq. (45). With the convention that $\prod_{a\in\varnothing}(\cdot)_a = 1$, the shifted moments μ_B can be expressed in terms of the central moments M_B via

$$\mu_B = \mathbb{E}\left[\prod_{a\in B}(V_a - I_a/G_a)\right] = \mathbb{E}\left[\prod_{a\in B}(V_a - m_a + \mu_a)\right] = \sum_{C \subset B} M_C \prod_{a \in B\setminus C} \mu_a ,$$

where we denote the first-order shifted moments $\mu_{\{a\}} = \mu_a$ for simplicity. Injecting the above relation into the recursive definition of the shifted moments

$$\alpha_B \mu_B = \sum_{C \subsetneq B} \gamma_{B;C} \mu_C ,$$

yields a recursive definition for the central moments. Specifically, we have

$$\begin{aligned} \alpha_B \left(M_B + \sum_{C\subsetneq B} M_C \prod_{a\in B\setminus C} \mu_a\right) &= \sum_{C\subsetneq B} \gamma_{B;C}\left(\sum_{D\subset C} M_D \prod_{a \in C\setminus D} \mu_a\right) . \\ &= \sum_{C\subsetneq B} M_C \left(\sum_{C\subset D\subsetneq B} \gamma_{B;D} \prod_{a\in D\setminus C} \mu_a\right) , \end{aligned}$$

so that the moment M_B can be obtained from the lower-order moments M_C, $C \subsetneq B$, via

$$M_B = \frac{1}{\alpha_B} \sum_{C\subsetneq B} M_C \left(\sum_{C\subset D\subsetneq B} \gamma_{B;D} \prod_{a\in D\setminus C} \mu_a - \alpha_B \prod_{a\in B\setminus C} \mu_a\right) , \tag{34}$$

with the convention that $M_\varnothing = 1$. Observe that for all singleton $\{a\}$, we consistently have $M_a = M_{\{a\}} = \mathbb{E}[V_a - m_a] = 0$. This follows from remembering that $\mu_a = \gamma_{a;\varnothing}/\alpha_a$ by Eq. (45), so that applying (34) for $B = \{a\}$ leads to

$$M_a = M_\varnothing \left(\gamma_{a;\varnothing} - \alpha_a \mu_a\right) = 0\,. \tag{35}$$

However it is hard to interpret (34) for higher order moments. To address this limitation, one can try to simplify (34) by writing the coefficients $\gamma_{B;D}$ featuring in (34) under a centered form:

$$\begin{aligned}
\gamma_{B;D} &= \mathbb{E}\left[\prod_{a\in D} Y_a \prod_{a\in B\setminus D} Q_a\left(1-Y_a\right)\right]\,,\\
&= \mathbb{E}\left[\prod_{a\in D} Y_a \prod_{a\in B\setminus D}\left(1-Y_a\right) \prod_{a\in B\setminus D}\left(R_a - m_a + \mu_a\right)\right]\,,\\
&= \sum_{E\subset B\setminus D}\left(\prod_{a\in E}\mu_a\right)\mathbb{E}\left[\prod_{a\in D} Y_a \prod_{a\in B\setminus D}\left(1-Y_a\right)\prod_{a\in (B\setminus D)\setminus E}\left(R_a - m_a\right)\right]\,.
\end{aligned}$$

It turns out that injecting the above centered form for $\gamma_{B;D}$ into Eq. (34) leads to a series of simplifications, which we give as intermediary results in S4 Appendix. Specifically, setting $F = B\setminus(E\cup D)$, for $C \neq \varnothing$, we show that

$$\begin{aligned}
\sum_{C\subset D\subsetneq B}\gamma_{B;D}\prod_{a\in D\setminus C}\mu_a = &-\left(\prod_{a\in B\setminus C}\mu_a\right)\mathbb{E}\left[\prod_{a\in B} Y_a\right] \\
&+ \sum_{F\subset B\setminus C}\left(\prod_{a\in B\setminus (C\cup F)}\mu_a\right)\mathbb{E}\left[\prod_{a\in C} Y_a \prod_{a\in F}\left(R_a - m_a\right)\left(1-Y_a\right)\right]
\end{aligned}\tag{36}$$

and for $C = \varnothing$, we show that

$$\begin{aligned}
\sum_{D\subsetneq B}\gamma_{B;D}\prod_{a\in D}\mu_a &= \left(\prod_{a\in B}\mu_a\right)\mathbb{E}\left[1-\prod_{a\in B} Y_a\right] \\
&\quad + \sum_{\varnothing\subsetneq F\subset B}\left(\prod_{a\in B\setminus F}\mu_a\right)\mathbb{E}\left[\prod_{a\in F}\left(R_a - m_a\right)\left(1-Y_a\right)\right]\,,\\
&= \alpha_B \prod_{a\in B}\mu_a \\
&\quad + \sum_{F\subset B, |F|>1}\left(\prod_{a\in B\setminus F}\mu_a\right)\mathbb{E}\left[\prod_{a\in F}\left(R_a - m_a\right)\left(1-Y_a\right)\right]\,.
\end{aligned}\tag{37}$$

where we have used Eq. (31) in the last equation. Using the above expressions will allow us to derive compact expressions for low-order centered moment in S5 Appendix.

S4 Appendix. Results for intermediary calculations. The goal of this appendix is to simplify the expression of the following quantity

$$\begin{aligned}
Q_C &= \sum_{C\subset D\subsetneq B}\gamma_{B;D}\prod_{a\in D\setminus C}\mu_a \\
&= \sum_{C\subset D\subsetneq B}\sum_{E\subset B\setminus D}\left(\prod_{a\in E\cup (D\setminus C)}\mu_a\right)\mathbb{E}\left[\prod_{a\in D} Y_a \prod_{a\in B\setminus D}\left(1-Y_a\right)\prod_{a\in (B\setminus D)\setminus E}\left(R_a - m_a\right)\right]\,.
\end{aligned}$$

Set $F = (B \setminus D) \setminus E = B \setminus (E \cup D)$, then F can be any set included in $B \setminus C$ and we have $E = B \setminus (F \cup D) = (B \setminus F) \setminus D$. Introducing F allows one to write the double sum over the sets D and E as a double sum over the set F and D. Specifically, for all set function f, if $C \neq \varnothing$, we have the following change of set variable

$$\sum_{C \subset D \subsetneq B} \sum_{E \subset B \setminus D} f(D, E) = \sum_{F \subset B \setminus C} \sum_{C \subset D \subset B \setminus F} f(D, (B \setminus F) \setminus D) - f(B, \varnothing)\,. \tag{38}$$

By direct evaluation, the boundary term for our case is found to be:

$$f(B, \varnothing) = \left(\prod_{a \in B \setminus C} \mu_a \right) \mathbb{E}\left[\prod_{a \in B} Y_a \right].$$

Since $(E \cup D) \setminus C = E \cup (D \setminus C) = B \setminus (C \cup F)$, the remaining sum $S_C = Q_C + f(B, \varnothing)$can be written as

$$S_C = \sum_{F \subset B \setminus C} \left(\prod_{a \in B \setminus (C \cup F)} \mu_a \right) S_{C,F} \tag{39}$$

where we have defined

$$S_{C,F} = \sum_{C \subset D \subset B \setminus F} \mathbb{E}\left[\prod_{a \in D} Y_a \prod_{a \in B \setminus D} (1 - Y_a) \prod_{a \in F} (R_a - m_a) \right].$$

The latter sum $S_{C,F}$ can be further evaluated by observing that

$$\begin{aligned}
S_{C,F} &= \sum_{C \subset D \subset B \setminus F} \mathbb{E}\left[\prod_{a \in D} Y_a \prod_{a \in B \setminus D} (1 - Y_a) \prod_{a \in F} (R_a - m_a) \right], \\
&= \mathbb{E}\left[\left(\sum_{C \subset D \subset B \setminus F} \prod_{a \in D} Y_a \prod_{a \in (B \setminus F) \setminus D} (1 - Y_a) \right) \prod_{a \in F} (R_a - m_a)(1 - Y_a) \right], \\
&= \mathbb{E}\left[\prod_{a \in C} Y_a \left(\sum_{H \subset B \setminus (F \cup C)} \prod_{a \in H} Y_a \prod_{a \in (B \setminus (F \cup C)) \setminus H} (1 - Y_a) \right) \prod_{a \in F} (R_a - m_a)(1 - Y_a) \right], \\
&= \mathbb{E}\left[\prod_{a \in C} Y_a \prod_{a \in F} (R_a - m_a)(1 - Y_a) \right],
\end{aligned}$$

where the last line follows from the binomial identity. Thus, we find that

$$\begin{aligned}
Q_C = &\sum_{F \subset B \setminus C} \left(\prod_{a \in B \setminus (C \cup F)} \mu_a \right) \mathbb{E}\left[\prod_{a \in C} Y_a \prod_{a \in F} (R_a - m_a)(1 - Y_a) \right] \\
&- \left(\prod_{a \in B \setminus C} \mu_a \right) \mathbb{E}\left[\prod_{a \in B} Y_a \right].
\end{aligned} \tag{40}$$

The above result can be further simplified for $C = \varnothing$, so that one ultimately obtain:

$$Q_\varnothing = \sum_{\varnothing \subsetneq F \subset B} \left(\prod_{a \in B \setminus F} \mu_a \right) \mathbb{E}\left[\prod_{a \in F} (R_a - m_a)(1 - Y_a) \right] + \left(\prod_{a \in B} \mu_a \right) \mathbb{E}\left[1 - \prod_{a \in B} Y_a \right].$$

These establishes the intermediary results Eq. (36) and Eq. (37) used in S3 Appendix.

S5 Appendix. Low-order mixed central moments formula. Consider two neurons indexed by a_1, a_2 in A and let us set $B = \{a_1, a_2\}$ in Eq. (34). As by definition of the centered moment, we have $M_a = M_{\{a\}} = 0$ for all a in A, we observe that there is only one nonzero term in Eq. (34) corresponding to $C = \varnothing$, so that

$$M_{a_1,a_2} = \frac{1}{\alpha_{a_1,a_2}} M_\varnothing \left(\sum_{D \subsetneq \{a_1,a_2\}} \gamma_{a_1,a_2;D} \prod_{a \in D} \mu_a - \alpha_{a_1,a_2} \prod_{a \in \{a_1,a_2\}} \mu_a \right) . \tag{41}$$

Using Eq. (37), we have

$$\sum_{D \subsetneq \{a_1,a_2\}} \gamma_{a_1,a_2;D} \prod_{a \in D} \mu_a = \alpha_{a_1,a_2} \prod_{a \in \{a_1,a_2\}} \mu_a + \mathbb{E}\left[\prod_{a \in \{a_1,a_2\}} (R_a - m_a)(1 - Y_a) \right] .$$

Injecting the above relation in Eq. (41) leads to the following compact expression for the centered second-order moments:

$$M_{a_1,a_2} = \frac{\mathbb{E}\left[(R_{a_1} - m_{a_1})(1 - Y_{a_1})(R_{a_2} - m_{a_2})(1 - Y_{a_2})\right]}{1/(b\tau_{a_1}) + 1/(b\tau_{a_2}) + \mathbb{E}\left[1 - Y_{a_1} Y_{a_2}\right]} .$$

For the case of three neurons $B = \{a_1, a_2, a_3\}$, the third-order centered moment can be expressed in terms of four coefficients, corresponding to choosing $C = \varnothing$, $\{a_1, a_2\}$, $\{a_2, a_3\}$, $\{a_1, a_3\}$ in formula Eq. (34) so that

$$\alpha_{a_1,a_2,a_3} M_{a_1,a_2,a_3} = M_\varnothing c_\varnothing + M_{a_1,a_2} c_{a_1,a_2} + M_{a_1,a_3} c_{a_1,a_3} + M_{a_2,a_3} c_{a_2,a_3} . \tag{42}$$

Using Eq. (37), we can write the zero-order coefficient $c_\varnothing$ as

$$\begin{aligned} c_\varnothing &= \mathbb{E}\left[\prod_{a \in \{a_1,a_2,a_3\}} (R_a - m_a)(1 - Y_a) \right] + \mu_{a_1} \mathbb{E}\left[\prod_{a \in \{a_2,a_3\}} (R_a - m_a)(1 - Y_a) \right] \\ &\quad + \mu_{a_2} \mathbb{E}\left[\prod_{a \in \{a_1,a_3\}} (R_a - m_a)(1 - Y_a) \right] + \mu_{a_3} \mathbb{E}\left[\prod_{a \in \{a_1,a_2\}} (R_a - m_a)(1 - Y_a) \right] , \\ &= \mathbb{E}\left[\prod_{a \in \{a_1,a_2,a_3\}} (R_a - m_a)(1 - Y_a) \right] \\ &\qquad + \mu_{a_1} \alpha_{a_2,a3} M_{a_2,a_3} + \mu_{a_2} \alpha_{a_1,a3} M_{a_1,a_3} + \mu_{a_3} \alpha_{a_1,a2} M_{a_1,a_2} , \end{aligned}$$

where we have utilized formula Eq. (42) for the second-order centered moments. Using Eq. (36), the second-order coefficient c_{a_1,a_2} can be written as

$$c_{a_1,a_2} = \gamma_{a_1,a_2,a_3;a_1,a_2} - \alpha_{a_1,a_2,a_3} \mu_{a_3} .$$

with

$$\gamma_{a_1,a_2,a_3;a_1,a_2} = \mu_{a_3} \mathbb{E}\left[Y_{a_1} Y_{a_2} (1 - Y_{a_3})\right] + \mathbb{E}\left[Y_{a_1} Y_{a_2} (R_{a_3} - m_{a_3})(1 - Y_{a_3})\right] . \tag{43}$$

Utilizing the definition of α_B given in Eq. (29), we have

$$\begin{aligned} c_{a_1,a_2} &= \mathbb{E}\left[Y_{a_1} Y_{a_2} (R_{a_3} - m_{a_3})(1 - Y_{a_3})\right] + \mu_{a_3} \left(\mathbb{E}\left[Y_{a_1} Y_{a_2} (1 - Y_{a_3})\right] - \alpha_{a_1,a_2,a_3} \right) , \\ &= \mathbb{E}\left[Y_{a_1} Y_{a_2} (R_{a_3} - m_{a_3})(1 - Y_{a_3})\right] - \mu_{a_3} \left(\frac{1}{b\tau_{a_3}} + \alpha_{a_1,a_2} \right) . \end{aligned}$$

Combining all the above results in Eq. (42) finally leads to the compact expression

$$\begin{aligned} &\alpha_{a_1,a_2,a_3} M_{a_1,a_2,a_3} = \\ &\qquad \mathbb{E}\left[(R_{a_1} - m_{a_1})(1 - Y_{a_1})(R_{a_2} - m_{a_2})(1 - Y_{a_2})(R_{a_3} - m_{a_3})(1 - Y_{a_3})\right] \\ &\qquad + \mathbb{E}\left[(R_{a_3} - m_{a_3})(1 - Y_{a_3})(Y_{a_1} Y_{a_2} - 1)\right] M_{a_1,a_2} \\ &\qquad + \mathbb{E}\left[(R_{a_2} - m_{a_2})(1 - Y_{a_2})(Y_{a_1} Y_{a_3} - 1)\right] M_{a_1,a_3} \\ &\qquad + \mathbb{E}\left[(R_{a_1} - m_{a_1})(1 - Y_{a_1})(Y_{a_2} Y_{a_3} - 1)\right] M_{a_2,a_3} . \end{aligned}$$

S6 Appendix. Event rates definitions and calculations. Let us consider a set of neuron B. By definition, each neuron $a \in B$ experiences a synaptic event whenever $W_a = W_{e,a} + W_{i,a} > 0$, where $W_{e,a}$ and $W_{i,a}$ denotes the random excitatory and inhibitory jumps delivered to neuron a. Let us denote the overall rate of synaptic events collectively experienced by the set of neuron B by b_B. Because neurons are allowed to receive synchronous, the rate b_B is generally strictly less than the sum of the event rates b_a experienced by each neuron $a \in B$ individually. However, the overall rate b_B can be expressed in terms of the individual rates b_a at the cost of introducing auxiliary conditional probabilities. To see this, one needs to first determine the event rate b_B in terms of the event rate of all subset $A \subsetneq B$. This can be done by remarking that

$$\begin{aligned} b_B &= b_B \mathbb{E}\left[\mathbb{1}_{\{\sum_{a\in B} W_a > 0\}}\right] , \\ &= b_B \mathbb{E}\left[\sum_{\varnothing \subsetneq A \subset B} (-1)^{|A|-1} \prod_{a\in A} \mathbb{1}_{\{W_a>0\}}\right] , \\ &= \sum_{\varnothing \subsetneq A \subset B} (-1)^{|A|-1} b_B \mathbb{E}\left[\prod_{a\in A} \mathbb{1}_{\{W_a>0\}}\right] , \\ &= \sum_{\varnothing \subsetneq A \subset B} (-1)^{|A|-1} b_A \mathbb{E}\left[\prod_{a\in A} \mathbb{1}_{\{W_a>0\}} \middle| \sum_{a\in B} W_a > 0\right] . \end{aligned} \quad (44)$$

Accordingly, let us denote the probability that all neurons $a \in A$ spikes given that a synaptic event occurs for B (i.e. at least one neuron $a \in B$ spikes) by

$$q_{A,B} = \mathbb{E}\left[\prod_{a\in A} \mathbb{1}_{\{W_a>0\}} \middle| \sum_{a\in B} W_a > 0\right] ,$$

and for simplicity, let us also denote $q_B = q_{B,B}$. The above probabilities are the auxiliary conditional probabilities required to specify b_B. Indeed, solving for b_B in Eq.(44), one obtains

$$b_B = \frac{1}{1+(-1)^{|B|} q_B} \sum_{\varnothing \subsetneq A \subsetneq B} (-1)^{|A|-1} b_A q_{A,B} ,$$

which upon iterated use yield an explicit formula specifying b_B in terms of b_a, $a \in B$:

$$b_B = \sum_{a\in B} b_a \sum_{m=1}^{|B|} \sum_{\{a\}=B_1 \subsetneq \ldots \subsetneq B_m = B} (-1)^{\sum_{k=1}^m |B_k| - m} \prod_{k=1}^m \left(\frac{q_{B_k,B_{k-1}}}{1+(-1)^{|B_m|} q_{B_m}}\right) .$$

The above formula and its derivation is perhaps easier to grasp for the case when B represents a pair of neurons, which we discuss now. To this end, let us denote $W_1 = W_{e,1} + W_{i,1}$ and $W_2 = W_{e,2} + W_{i,2}$, then we have

$$\begin{aligned} b_{12} &= b_{12} \mathbb{E}\left[\mathbb{1}_{\{W_1+W_2>0\}}\right] , \\ &= b_{12} \mathbb{E}\left[\mathbb{1}_{\{W_1>0\}} + \mathbb{1}_{\{W_2>0\}} - \mathbb{1}_{\{W_1>0, W_2>0\}}\right] , \\ &= b_1 + b_2 - b_{12} q_{12} , \end{aligned}$$

where we have defined $q_{12} = \mathbb{P}\left[W_1 > 0, W_2 > 0 \,|\, W_1 + W_2 > 0\right]$. This directly implies that

$$b_{12} = \frac{b_1 + b_2}{1 + q_{12}} .$$

One can thus check that when inputs to neuron 1 and neuron 2 are independent, one has $q_{12} = 0$, so that one obtains the upper bound $b_{12} = b_1 + b_2$ as expected. By contrast, when neurons are perfectly synchronous, so that the neuron with minimum input rate $\min(b_1, b_2)$, only receives inputs when the neurons with maximum input rate $\max(b_1, b_2)$ also receives an input, one has $q_{12} = \min(b_1, b_2)/\max(b_1, b_2)$. Thus, one obtains the lower bound

$$b_{12} = \frac{b_1 + b_2}{1 + \min(b_1, b_2)/\max(b_1, b_2)} = \max(b_1, b_2) ,$$

as expected.
